# Stent revascularization versus bypass surgery for peripheral artery disease in type 2 diabetic patients – an instrumental variable analysis

**DOI:** 10.1038/srep37177

**Published:** 2016-11-18

**Authors:** Chia-Hsuin Chang, Jou-Wei Lin, Jiun Hsu, Li-Chiu Wu, Mei-Shu Lai

**Affiliations:** 1 Department of Internal Medicine, National Taiwan University Hospital, Taipei, Taiwan; 2 Department of Medicine, College of Medicine, National Taiwan University, Taipei, Taiwan; 3 Institute of Epidemiology and Preventive Medicine, College of Public Health, National Taiwan University, Taipei, Taiwan; 4 Cardiovascular Center, National Taiwan University Hospital Yunlin Branch, Douliou City, Yunlin County, Taiwan; 5 Department of Surgery, National Taiwan University Hospital Yunlin Branch, Douliou City, Yunlin County, Taiwan

## Abstract

The objective of this study was to use instrumental variable (IV) analyses to evaluate the clinical effectiveness of percutaneous stent revascularization versus bypass surgery in the treatment of peripheral artery disease (PAD) among type 2 diabetic patients. Type 2 diabetic patients who received peripheral artery bypass surgery (n = 5,652) or stent revascularization (n = 659) for lower extremity arterial stenosis between 2000 and 2007 were identified from the Taiwan National Health Insurance claims database. Patients were followed from the date of index hospitalization for 2 years for lower-extremity amputation, revascularization, and hospitalization for medical treatment. Analysis using treatment year, patients’ monthly income level, and regional difference as IVs were conducted to reduce unobserved treatment selection bias. The crude analysis showed a statistically significant risk reduction in favor of stent placement in lower extremity amputation and in the composite endpoint of amputation, revascularization, or hospitalization for medical treatment. However, peripheral artery stent revascularization and bypass surgery had similar risk of lower limb amputation and composite endpoints in the analyses using calendar year or patients’ monthly income level as IVs. These two treatment modalities had similar risk of lower limb amputation among DM patients with PAD.

Peripheral artery disease (PAD), which results from progressive narrowing of arteries secondary to atherosclerosis, has increased in prevalence and put a severe burden on the patients as well as the economy^1^,^2^. Patients with type 2 diabetes mellitus (DM) are at increased risk of developing lower extremity PAD^3^,^4^. Diabetic patients with PAD commonly show involvement of the arteries below the knee, especially at the tibial and peroneal arteries, and involvement of the profunda femoris^5^. Although diabetic patients with PAD have similar primary patency rates, restenosis, and secondary patency rates compared to non-diabetic patients, the mortality and amputation rates are markedly higher in DM patients^6^,^7^.

Endovascular revascularization and open bypass surgery are the two strategies for managing PAD patients with disabling claudication after medical therapy has failed to improve symptoms and those with critical limb ischemia^8^,^9^. The choice of a procedure depends on many factors, such as site and extent of the disease, distal run off and surgical risk due to associated cardiovascular disease^6^,^10^. Proximal, short segment disease in the iliac and femoral segments is amenable to percutaneous transluminal angioplasty (PTA). Studies suggested that for the treatment of focal iliac stenosis and occlusion, PTA with or without stenting has become a viable alternative to surgical reconstruction due to its safety and acceptable long-term outcomes^11^,^12^,^13^. Recent guidelines even suggest an “endovascular first” approach when revascularization is indicated^14^.

Controversies exist as which one is the better treatment modality for more distal disease in the popliteal and tibial arteries^15^,^16^. Patients with more advanced and extensive occlusive disease is better managed by bypass grafting; however, the procedure is hindered by complications that result in severe morbidity and excessive costs. PTA below the knee is recommended to patients with critical limb ischemia who are at high risk during surgical revascularization because of comorbidities. It may achieve satisfactory limb salvages rates at least in the short-term. However, regarding the distal and diffuse nature of the disease in diabetic patients, PTA are technically difficult to perform^6^. In particular, little is known regarding the comparative effectiveness of stent revascularization versus bypass surgery, and currently available literature suggests that there is probably no difference in the clinical outcome^17^. To our knowledge, no randomized trial comparing the efficacy between endovascular stenting and bypass surgery in diabetic patients with lower extremity PAD has been published. Despite an increasing number of DM patients received stent revascularization for lower extremity arterial occlusive disease, a large-scale study comparing the clinical effectiveness in limb salvage between those who underwent percutaneous stent revascularization or bypass surgery for more distal lower extremity arterial disease in real clinical setting is still lacking^18^. Because patients were selected for treatment modality primarily based on the anatomy and extent of the disease, and the detailed information on vascular anatomy was not available in most databases, traditional multivariable regression analysis controlling for only measured and recorded variables may not provide valid results. Compared with standard modeling, instrumental variable (IV) analysis may produce less biased estimates of treatment effect in the setting that unmeasured patients characteristics affect both the decision to treat the the outcome^19^,^20^. In this study, we conducted IV analyses to reduce potential treatment selection bias and to compare the results from analyses using different IVs.

## Results

**Figure 1:**
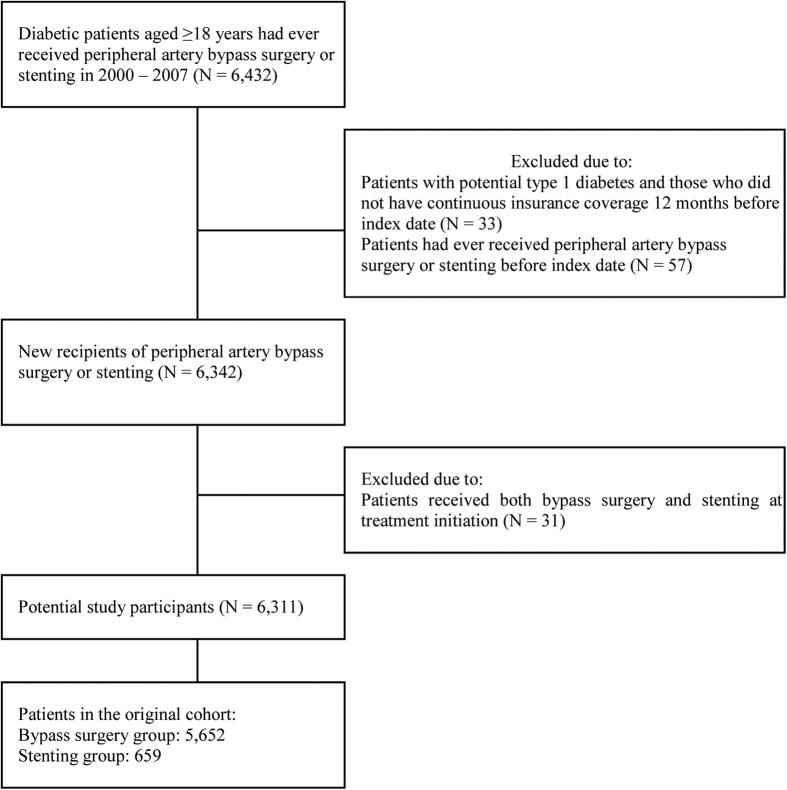
Study flow.

A total of 6,342 new patients receiving endovascular stents or bypass surgery for lower extremity arterial stenosis were identified during the study period. After exclusion of patients receiving both bypass surgery and stents in the same treatment episode, a total of 659 and 5,652 type 2 diabetic patients received peripheral artery stents and bypass surgery, respectively (Fig. 1). As identified in Table 1, the two treatment groups differed in a number of baseline characteristics. Patients receiving stenting were older, with higher proportion of male, more likely to have ischemic heart and cerebrovascular disease, and receiving oral diabetic agents, aspirin, clopidogrel, cilostazol, statins, and prior history of coronary revascularization. In contrast, patients receiving bypass surgery were more likely to have retinopathy, nephropathy, and chronic renal disease, and insulin therapy. Higher proportion of the patients had prior history of lower extremity amputation, hospitalization for diabetes, and lower extremity complications.

Tables 2, 3, 4 presented the empirical data about the instrumental conditions associated with each study instrument^21^. The odds ratio between instruments and endovascular stenting as the measurement of strength of association was 4.79 for calendar year 2006–2007 (Table 2), 1.54 for monthly income level ≤17,280 NTD (Table 3), and 2.18 for the hospital located in northern Taiwan as a measure of regional difference (Table 4). The baseline data and mean predicted probability of lower limb amputation between the early (2003–2005) and late (2006–2007) treatment groups were shown in Table 2. Baseline demographics, co-morbidities, medication use, resource utilization, and amputation rates were similar between two groups, although a higher proportion of patients in the early treatment group received biguanides, sulfonylurea, clopidogrel, and underwent amputation at the index hospitalization. Despite of these small differences, the variables assumed as proxies of arterial stenosis severity (the proportion of those with prior amputation, the mean number of cardiovascular surgeon outpatient visits, and the mean number of hospitalizations due to lower extremity complications) were similar between the early and late periods. Meanwhile, the predicted probability of lower extremity amputation after initial management, our outcome of interest, was also similar between two groups (Table 2). Meanwhile, although peripheral artery stent recipients with monthly income level ≤17,280 NTD were older and more likely to have cardiovascular and cerebrovascular diseases but less likely to have chronic renal disease as compared with those with monthly income level >17,280 NTD, the mean predicted probability of amputation was similar between two groups (Table 3). These findings support the use of calendar year and perhaps patients’ monthly income level as a valid instrumental variable. In contrast, patients receiving peripheral artery stenting at non-northern hospitals had positive association with some risk factors of the outcome, for example, neuropathy and chronic renal disease, and had a significantly higher probability of lower limb amputation, raising the concerns that the regional difference as measured by the hospital location might also be associated with other unmeasured confounders (Table 4). The proportion of compliers in the analyses using calendar time, monthly income level, and regional difference as IV was 0.74, 0.45, and 0.58, respectively.

The crude analysis showed a statistically significant risk reduction in favor of stent placement in lower extremity amputation and in the composite endpoint of amputation, revascularization, or hospitalization for medical treatment (Table 5). However, peripheral artery stent revascularization and bypass surgery had similar risk of lower limb amputation and composite endpoints in the analyses using calendar year or patients’ monthly income level as IV. Risk difference between two treatment modalities was −0.003 (95% CI: −0.053~0.046; *p* = 0.89) for lower limb amputation and −0.027 (95% CI: −0.080~0.026; *p* = 0.31) for the composite endpoint of amputation, revascularization, or hospitalization for medical treatment while using calendar year as IV; and the risk difference was 0.058 (95% CI: −0.055~0.171; *p* = 0.31) and 0.016 (95% CI: −0.105~0.137; *p* = 0.79) for the two outcomes, respectively, in the analysis using monthly income level as IV (Table 5). In contrast, stent revascularization was associated with a significantly higher risk of amputation and composite endpoint in the analysis using regional difference as IV. This set of estimators were biased as regional difference did not perfectly meet the instrumental conditions (see above).

## Discussion

The results of this study demonstrated that, among type 2 diabetic patients, percutaneous stent revascularization for lower extremity arterial stenosis is not superior to open bypass surgery in reducing lower limb amputation rates during the first two years following the procedure. The stent-based endovascular procedure was also found to result in a similar risk of the composite endpoint consisting of amputation, revascularization, or hospitalization for medical treatment.

Rapidly evolving technology in stent procedure causes tremendous changes in clinical practice that occur so quickly even before the randomized controlled trials can be completed. The most current guidelines regarding the revascularization treatment of lower extremity PAD continue to be based upon the implications from the randomized BASIL (Bypass Versus Angioplasty in Severe Ischemia of the Leg) trial published several years ago, which suggests similar amputation-free survival with a bypass-surgery-first and a balloon-angioplasty-first strategy in patients with severe limb ischemia due to infra-inguinal disease^22^,^23^,^24^. Constrained by the insufficient evidence due to limited data, further studies to confirm or refute these findings and recommendations are urgently required as there was a substantial increase in the number of patients receiving lower extremity percutaneous stent revascularization during the past decade^25^,^26^. Analyzing observational data may provide a timely evaluation for medical device safety and effectiveness in the real-world settings. However, substantial challenges exit particularly while using non-experimental design to examine comparative effectiveness and safety between open surgery and stent revascularization. In order to make a valid comparison between treatment groups, important information such as patients’ surgical risks, symptoms, detailed vascular anatomy, presence and severity of underlying diseases, concomitant medications, and lifestyle factors were needed to be collected and taken into consideration. Even in the comprehensive registry data, these variables were incompletely measured and recorded such that residual confounding still persisted by using conventional multivariable regression approach. Therefore, research methodology that can handle unmeasured confounding is particularly useful in this setting. Our study results from an instrumental variable analysis which assumed to provide valid estimates even without measuring the confounders were consistent with the conclusion from the BASIL trial^22^. Our study findings suggested that instrumental variable analysis might effectively reduce confounding by indication, which was a more serious problem in the setting of non-pharmacological therapy as compared with pharmacological treatment.

In the present study, we conducted analyses utilizing several potential instruments. Although treatment calendar year and patients’ income level might conceptually meet the required assumptions, it was more appropriate to use calendar year as the instrument based on empirical data because it had a higher strength of association with treatment and there was evidence that it was not associated with measured confounders nor the outcome. Results from analysis using patients’ monthly income level as the instrument did not substantially change conclusion, although monthly income level had a lower association with treatment and there was some association with certain measured proxy for the severity of lower-extremity vascular occlusion. It was surprisingly shown that patients’ characteristics and perhaps disease severity varied among different regions. Meanwhile, it is possible that the regional difference in operators’ experiences can directly influence patients’ outcome. Our study findings suggested that using an instrumental variable that was associated with unmeasured poor prognostic factors for the outcome might “over-correct” the true association between exposure of interest and outcome. Further simulation studies are required to quantitatively evaluate the direction and magnitude of bias when there was a relation between the instrument and measured confounders^21^.

This study has some limitations that warrant mention. First, the use of data from a national claims database prevented us from distinguishing between inflow revascularization procedures (aortoiliac) and infrainguinal (femoropopliteal) procedures. Although diabetic patients with PAD commonly had arterial occlusion below the knee, it is still conceivable that stenting could have been performed more commonly than surgery for those with iliac disease, where more favorable outcomes would be expected. In contrast, smoking, obesity, and body height, despite that they were risk factors for lower limb arterial stenosis and amputation^27^,^28^,^29^, did not confound study results as they were not associated with treatment decision. Second, as endovascular intervention for peripheral artery stenosis was relatively a new medical technology as compared with open bypass surgery during the study period, we could not exclude the possibility that the safety and effectiveness of stent revascularization may improve after operators become more experienced. Third, as the instrumental analysis only provided marginal effect estimates, we did not further explore whether the comparative effectivenss of stenting versus surgery may be different in certain risk groups. Fourth, we excluded the cases who received balloon angioplasty only and who received both stent revascaularization and bypass surgery. Accordingly, we remain conservative in generalizing our results to strategies other than the stent-first and surgery-first approach. Finally, since the follow-up duration was only 2.0 years, a longer time is required to observe the long-term effects of different revascularization strategies.

The evaluation of clinical outcomes between stent and surgery for PAD could be biased if the data were obtained from a direct comparison of two “unbalanced” groups in a claims database. The instrumental variable behaves like a natural randomization if it is highly correlated with treatment, but not related to patient characteristics or other confounding factors, nor directly affecting the outcome of interest. Treatment calendar year and patients’ income level meet the required assumptions of good instrumental variables. In conclusion, this quasi-experimental study showed that percutaneous stent revascularization was associated with a similar risk of lower limb amputation as compared with open bypass surgery for type 2 diabetic patients with PAD. Although these results can inform a potential revision of the recommendations for PAD treatment, further randomized clinical trials are warranted.

## Methods

### Data Source

Taiwan’s National Health Insurance (NHI) database includes complete outpatient visits, hospital admissions, prescriptions, disease and vital status for 99% of the country’s population (approximately 23 million) in Taiwan. The claims datasets were linked with the National Death Registry through the use of birth dates and civil identification numbers unique to each beneficiary. The protocol of this study was approved by the National Taiwan University Hospital Research Ethics Committee. Informed consent was waived due to the retrospective and anonymous nature of the claims data. All analyses were performed in accordance with relevant guidelines and regulations.

### Diabetic Cohort

All patients’ records tagged with a diabetes diagnosis code (Supplementary Table 1) between 1 January 2000 and 31 December 2000 were retrieved from the claims database. An algorithm demonstrated a high level of sensitivity and positive predictive value (93.2% and 92.3% respectively) for identifying DM patients^30^. A total of 640,173 patients was identified using the algorithm. Patients were followed from the date of DM diagnosis in 2000 until death, disenrollment from the national health insurance, or the end of the study period (31 December 2008).

### Study Population and Comparison Groups

Using services or procedure claims documented between 1 January 2000 and 31 December 2007, we identified patients who received peripheral artery bypass surgery or stent placement for lower extremity arterial stenosis. The date of hospitalization was defined as the index date. Four types of percutaneous stents were approved for treating peripheral arterial stenosis in Taiwan during the study period (Supplementary Table 2). For those who had two or more hospitalizations for the procedure, the date of the first hospitalization was defined as the index date. We further excluded patients with type 1 DM. Also excluded were patients who did not have continuous insurance coverage for 12 months before the procedure, those who received peripheral artery bypass surgery or stent placement before the year 2000, and those who received both peripheral artery bypass surgery and stent placement during the same hospitalization.

### Outcome Definition

The primary outcome of this study was lower extremity amputation, defined by health insurance procedure claims from the inpatient dataset. Lower limb amputation was further stratified by site (i.e., thigh, leg, ankle, and foot). The secondary outcome was a composite endpoint of lower limb amputation, revascularization, and hospitalization for medical treatment including prostaglandin E1, anti-coagulant, or thrombolytic therapy.

### Covariate Ascertainment and Adjustment

Inpatient and outpatient diagnosis files as well as the prescription files during the 12-month period before the index date were used to ascertain patients’ past history (ICD-9-CM codes provided in Supplementary Table 1). The medication of each patient during this period was also ascertained (Supplementary Table 3).

### Statistical Analysis

The instrumental variable (IV) behaves like a natural randomization if it is highly correlated with treatment, but not related to patient characteristics or other confounding factors, nor directly affecting the outcome of interest^31^,^32^. Prior studies suggested that treatment calendar year can be used as an IV, given a higher proportion of patients receiving stent placements over time when other patient characteristics were similar^33^. Other potential IVs included geographical or regional difference and physician preference as they may also meet the instrumental conditions^34^. Furthermore, in certain circumstance, suppose patients’ income substantially influence their treatment decisions due to they have to pay out-of-pocket for the treatment, but is not related to other confounding factors, then it may be appropriate to use recipients’ income as an IV. In the present study, we compared results from analyses using different IVs by dividing all patients receiving peripheral artery bypass surgery or stent placement into 1) the early (2003–2005) or late (2006–2007) group based on treatment year; 2) low (≤17,280 New Taiwan Dollar, NTD) or high (>17,280 NTD) monthly income level; and 3) regional difference as measured by the hospital location in the northern or non-northern parts of Taiwan. We did not use doctors’ preference as an instrument because cardiologists can only perform stenting while bypass surgery can only be performed by cardiovascular surgeons in Taiwan (treatment is almost 100% correlated with doctors’ specialty).

We followed the recommendations suggested by Swanson SA *et al*. in conducting instrumental variable analyses^21^. First, we calculated the odds ratios as the strength of association between the proposed instruments and the treatment. Second, falsification tests were done to examine differences in baseline characteristics and the probability to develop outcomes of interest across the levels of instruments. With a logistic regression model, we estimated the the probability of developing study endpoints using indicators for stent placement, as well as age, sex, treatment year, underlying diseases, concomitant medications, 12 months prior to the index date. Third, because we intended to evaluate the comparative effectiveness of endovascular stenting versus bypass sugery in the subpopulation of “compliers”, the proportion of the study population composed of compliers was estimated. If the above conditions appeared justifiable, we then performed a 2-stage least-squares regression to calculate the marginal IV effect estimate. The first stage predicted the stent placement (treatment) as a linear function of IV and all other observed variables. In the second stage, outcome of interest was regressed on the predicted probability of stent placement (treatment), derived from the first stage, along with covariates that were significantly imbalanced between the comparison groups. The regression coefficient for the predicted probability of stent placement (treatment) can be interpreted as an unbiased estimate for risk difference of peripheral artery stenting relative to bypass surgery. More details about instrumental variables were explained in “The Methods of Instrumental Variables.”

All statistical analyses were performed with SAS 9.2 (SAS Institute, Cary, NC). Two-tailed p values less than 0.05 were regarded as statistically significant.

### The Methods of Instrumental Variables

Instrumental variable (IV) analysis has primarily been used in economics and social science research to control for confounding and measurement error. Unlike propensity scores, IV can adjust for both measured and unmeasured confounding effects, and hence, allows for the possibility of making causal inferences with observational data. IV is under-used in medical research because it is not well-known to many researchers, and very few good IVs can be found in real clinical setting. In the published medical literature, geographical or regional difference, physician preference, and treatment calendar year have been used as good IVs in the claims database study to reduce confounding effect by unmeasured important clinical factors that are not amenable by traditional regression or propensity score analysis^35^,^36^,^37^,^38^,^39^.

There are three requirements for a variable to be chosen as a good IV (Supplementary Figure 1) the instrument is associated with the treatment, 2) the instrument does not affect the outcome except through treatment, and 3) the instrument does not share any share any cause with the outcome. A perfect example is the randomization indicator in a randomized controlled trial, which is more familiar to medical research readers. More specifically, 1) randomization itself is associated with the treatment, 2) randomization affects the outcome only through experimental treatment, and 3) randomization will create two comparable groups that were very similar in terms of measured and unmeasured risk factors. In observational study, if a good IV can be identified, then IV analysis can be applied to get an unbiased estimate of treatment effect. However, we cannot “prove” a variable to be a good instrument in non-experimental study design, but can only use subject-matter knowledge and provide some empirical data in support of a variable meeting the above three instrumental conditions. The aim of this study is to compare three potential IVs (treatment calendar time, monthly income level, and regional difference) and to use IV analysis evaluating the benefit of stent versus bypass surgery that is closer to the true effect.

For condition #1, an odds ratio between IV and treatment is calculated to evaluate the strength of association. For condition #2 and #3, we present data that showing the proposed instrument is not associated with measured confounders. More specifically, when study participants were categorized based on calendar year (IV), there was an equal distribution in all potential risk factors for the outcome (amputation, revascularization, or hospitalization for medical treatment) (Table 2), just like the first Table in a randomized controlled trial report to demonstrate comparability between treatment groups. This condition also hold true for monthly income level (Table 3), but not for region (Table 4), suggesting calendar year and monthly income level are more appropriate IVs than regional difference.

In a two-arm randomized placebo-controlled trial example, a patient randomized to the treatment group may actually not receive active treatment and a patient randomized to the placebo group may eventually receive active treatment. The compliers are the subset of the trial participants who comply with treatment assigned, that is, the total numbers of participants receive active treatment in the assigned treatment group plus numbers of participants receive placebo in the assigned placebo group then divided by the total number of study participants. This is an important information as the estimated treatment effect will be influenced by the proportion of compliers in a randomized trial. For example, if the true treatment effect is to reduce the risk by 80%, we can only observe a 40% reduction of the risk in a trial with 50% compliers. In statistical language, there are two kinds of treatment effect that can be evaluated, the average treatment effect in the total population (assuming homogeneity, that is, similar effects for all participants) and the local average treatment effect (LATE) in the subpopulation of compliers (assuming non-homogeneity, that is, not all participants had similar effects). Here, the LATE is a treatment effect in a subset of study population, that is, compliers, for whom would have this effect. In the above example, it is very natural to assume the observed treatment effect applies only to those who comply with the trial protocol. IV analysis requires the fourth assumption that the instrument only affects the treatment in one direction (monotonicity). That is, no one takes the opposite of the assigned treatment (“defiers”). Under monotonicity assumption, the IV estimator is consistent with LATE for the compliers. Similar to a randomized placebo-controlled trial example, in our study, for calendar year as an IV, the proportion of compliers would be the number of patients receiving stenting in 2006–2007 plus the number of patients receiving bypass surgery in 2003–2005 then divided by the total number of patients receiving bypass surgery and stenting in 2003–2007 = 396 + 4301/6311 = 0.74. The proportion of compliers in the analyses using monthly income level and regional difference as IV was 0.45, and 0.58, respectively, also suggesting that using treatment calendar year as an IV was more appropriate.

In order to support our argument that IV analysis is superior to traditional or commonly used propensity score analysis to get effect estimates more close to the truth, we also created a propensity-score matched cohort of stent revascularization (n = 433) versus peripheral artery bypass surgery (n = 1,196) for the planned comparison (Supplementary Table 4). A significant decrease in amputation risk was found for patients receiving stent revascularization (adjusted HR: 0.67; 95% CI: 0.48–0.92) (Supplementary Table 5). These results, contradictory to the findings from the randomized BASIL (Bypass Versus Angioplasty in Severe Ischemia of the Leg) trial showing similar amputation-free survival with a bypass-surgery-first and a balloon-angioplasty-first strategy, suggesting that IV analysis may be a more appropriate design in analyzing real world data in examining the effectiveness and safety of stent vascularization.

## Additional Information

**How to cite this article**: Chang, C.-H. *et al*. Stent revascularization versus bypass surgery for peripheral artery disease in type 2 diabetic patients – an instrumental variable analysis. *Sci. Rep*. **6**, 37177; doi: 10.1038/srep37177 (2016).

**Publisher’s note**: Springer Nature remains neutral with regard to jurisdictional claims in published maps and institutional affiliations.

## Supplementary Material

Supplementary Information

## Figures and Tables

**Table 1:** Baseline characteristics of type 2 diabetic patients with lower extremity artery stenosis during the 12-month period preceding peripheral artery bypass surgery or stenting.

	Original cohort		
	Bypass surgery (N = 5,652)	Stenting (N = 659)	P values
Age (mean ± SD)	67.96 ± 10.12	71.20 ± 8.68	<0.001
Male (%)	48.53	66.46	<0.001
Calendar year (%)			
2000–2002	39.97	0.00	<0.001
2003–2005	36.13	39.91	
2006–2007	23.90	60.09	
Monthly income in New Taiwan Dollar (%)			
≤17,280	58.10	68.13	<0.001
17,281~22,800	34.52	23.37	
22,801~28,800	2.34	2.28	
28,801~36,300	1.86	2.73	
36,301~45,800	1.70	1.52	
>45,800	1.49	1.97	
Region (%)			
Northern	42.52	61.76	<0.001
Central and eastern	22.08	17.15	
Southern	35.40	21.09	
Hospitalization department (%)			
Cardiologist	4.14	52.96	<0.001
Other	45.68	37.03	
Cardiovascular surgeon	50.18	10.02	
*Comorbidities* (*%*)			
Cardiovascular disease	90.15	97.42	<0.001
Ischemic heart disease	45.24	64.19	<0.001
Cerebrovascular disease	28.95	55.69	<0.001
Ketoacidosis or hyperosmolarity	3.79	1.97	0.018
Retinopathy	30.52	29.29	0.515
Neuropathy	34.34	28.53	0.003
Nephropathy	73.66	50.08	<0.001
Myocardial infarction	6.51	7.44	0.366
Ischemic stroke	17.85	46.74	<0.001
Chronic renal failure	53.47	22.15	<0.001
Chronic liver disease	11.31	10.47	0.520
Chronic lung disease	22.52	22.46	0.970
Depression	5.68	5.77	0.927
Charlson’s index (mean ± SD)	4.88 ± 2.36	4.09 ± 2.26	<0.001
Number of different ICD-9 diagnoses (mean ± SD)	21.68 ± 9.78	21.34 ± 9.80	0.402
Number of cardiovascular-related diagnoses (mean ± SD)	3.50 ± 2.81	4.78 ± 3.12	<0.001
*Medication use* (*%*)			
Biguanides	44.37	62.97	<0.001
Sulfonylurea	61.92	72.53	<0.001
Alpha-glucosidase inhibitors	11.48	19.73	<0.001
Thiazolidinediones	11.91	20.94	<0.001
Glinides	15.99	18.06	0.174
Any oral anti-diabetic agents	72.08	83.46	<0.001
Fast-acting insulins	49.91	32.32	<0.001
Basal insulins	16.54	11.53	0.001
Aspirin	64.23	78.15	<0.001
Clopidogrel	12.85	44.76	<0.001
Cilastazol	15.34	23.07	<0.001
Warfarin	7.02	7.28	0.805
ACE inibitors	45.67	42.03	0.076
Angiotensin receptor blockers	34.68	52.20	<0.001
Alpha-blockers	17.37	22.61	0.001
Beta-blockers	50.50	62.37	<0.001
Calcium channel blockers	74.45	76.48	0.257
Directics	58.09	55.99	0.303
Other anti-hypertensive agents	12.95	7.74	<0.001
Statins	28.34	52.50	<0.001
Fibrates	12.38	15.33	0.032
Number of different prescription drugs (mean ± SD)	49.90 ± 27.02	45.83 ± 23.39	<0.001
Number of cardiovascular-related medications (mean ± SD)	7.46 ± 5.34	8.04 ± 4.91	0.004
*Resource utilization* (*mean* ± *SD*)			
Number of A_1_C measurement	1.34 ± 1.61	2.07 ± 1.88	<0.001
Number of lipid-related lab test	4.35 ± 5.05	5.64 ± 5.04	<0.001
Number of peripheral artery ultrasound examination	0.03 ± 0.23	0.03 ± 0.21	0.839
Number of outpatient visits due to diabetes	15.76 ± 11.25	18.08 ± 12.09	<0.001
Number of outpatient visits not due to diabetes	46.06 ± 24.97	50.65 ± 28.98	<0.001
Number of emergency department visit	1.94 ± 2.73	1.48 ± 2.56	<0.001
Number of cardiology outpatient visits	7.12 ± 10.19	5.70 ± 8.87	<0.001
Number of cardiovascular surgery outpatient visits	3.55 ± 6.55	2.42 ± 5.11	<0.001
Number of cardiovascular-related physician visits	13.18 ± 11.02	19.51 ± 12.36	<0.001
Coronary revascularization %	3.18	10.93	<0.001
Prior amputation %	11.23	4.10	<0.001
Amputation at index hospitalization %	17.00	2.43	<0.001
Number of hospitalization due to diabetes	1.68 ± 1.81	1.00 ± 1.38	<0.001
Number of hospitalization not due to diabetes	2.09 ± 2.14	1.27 ± 1.59	<0.001
Number of hospitalization due to lower extremity complications	0.42 ± 0.85	0.13 ± 0.44	<0.001
Number of hospital days	23.29 ± 34.96	11.62 ± 24.22	<0.001
Number of cardiovascular-related hospital days	1.32 ± 1.66	1.07 ± 1.40	<0.001
Outcome occurrence (%)			
Amputation	15.61	7.28	<0.001
Thigh	2.51	1.06	0.020
Foot, ankle, leg	11.71	5.92	<0.001
Amputation, revascularization, or hospitalization for medical treatment	18.88	8.50	<0.001

SD: standard deviation.

**Table 2:** Baseline characteristics and clinical outcomes of diabetic patients with lower extremity arterial stenosis receiving peripheral artery stenting by calendar year.

	2003–2005 (N = 263)	2006–2007 (N = 396)	P-value
Age (mean ± SD)	70.44 ± 8.34	71.71 ± 8.88	0.066
Male (%)	67.68	65.66	0.590
*Comorbidities*			
Diabetes-related late complications (%)			
Cardiovascular disease	98.48	96.72	0.162
Ischemic heart disease	64.64	63.89	0.844
Cerebrovascular disease	61.22	52.02	0.020
Ketoacidosis or hyperosmolarity	2.28	1.77	0.642
Retinopathy	30.42	28.54	0.603
Neuropathy	27.76	29.04	0.721
Nephropathy	46.77	52.27	0.166
Myocardial infarction	8.75	6.57	0.296
Ischemic stroke	51.33	43.69	0.054
Chronic renal failure	18.63	24.49	0.076
Chronic liver disease	11.41	9.85	0.522
Chronic lung disease	26.62	19.70	0.037
Depression	5.32	6.06	0.691
Charlson’s index (mean ± SD)	4.10 ± 2.34	4.09 ± 2.21	0.982
Number of different ICD-9 diagnoses (mean ± SD)	21.46 ± 10.16	21.26 ± 9.57	0.800
Number of cardiovascular-related diagnoses (mean ± SD)	5.11 ± 3.10	4.56 ± 3.13	0.027
*Medication use* (*%*)			
Biguanides	69.96	58.33	0.002
Sulfonylurea	77.95	68.94	0.011
Alpha-glucosidase inhibitors	16.73	21.72	0.115
Thiazolidinediones	22.05	20.20	0.567
Glinides	19.77	16.92	0.351
Fast-acting insulins	31.18	33.08	0.609
Basal insulins	12.93	10.61	0.361
Aspirin	80.61	76.52	0.213
Clopidogrel	50.19	41.16	0.022
Cilostazol	19.39	25.51	0.068
Warfarin	9.13	6.06	0.138
ACE inibitors	43.73	40.91	0.473
Angiotensin receptor blockers	56.65	49.24	0.062
Alpha-blockers	24.33	21.46	0.388
Beta-blockers	64.26	61.11	0.414
Calcium channel blockers	78.71	75.00	0.272
Directics	59.70	53.54	0.119
Statins	52.85	52.27	0.884
Fibrates	14.45	15.91	0.610
*Resource utilization* (*mean* ± *SD*)			
Number of A_1_C measurement	2.00 ± 1.87	2.11 ± 1.89	0.448
Number of lipid-related lab test	5.75 ± 5.35	5.57 ± 4.83	0.657
Number of peripheral artery ultrasound examination	0.02 ± 0.12	0.04 ± 0.25	0.058
Number of outpatient visits due to diabetes	19.04 ± 12.26	17.45 ± 11.95	0.099
Number of outpatient visits not due to diabetes	50.90 ± 31.05	50.49 ± 27.56	0.863
Number of emergency department visit	1.33 ± 2.22	1.58 ± 2.76	0.195
Number of cardiology outpatient visits	5.69 ± 9.13	5.70 ± 8.70	0.982
Number of cardiovascular surgeon outpatient visits	2.87 ± 6.29	2.13 ± 4.14	0.091
Number of cardiovascular-related physician visits	20.08 ± 11.96	19.13 ± 12.63	0.335
Coronary revascularization %	9.89	11.62	0.486
Prior amputation %	3.04	4.80	0.265
Amputation at index hospitalization %	*	*	0.023
Number of hospitalization due to diabetes	1.08 ± 1.49	0.94 ± 1.29	0.244
Number of hospitalization not due to diabetes	1.33 ± 1.65	1.23 ± 1.55	0.438
Number of hospitalization due to lower extremity complications	0.15 ± 0.50	0.12 ± 0.39	0.400
Number of hospital days	11.67 ± 20.75	11.59 ± 26.30	0.967
Number of cardiovascular-related hospital days	1.11 ± 1.42	1.04 ± 1.38	0.552
Outcome occurrence (%)			
Amputation	6.08	8.08	0.334
Thigh	1.14	1.01	0.999
Foot, ankle, leg	4.94	6.57	0.387
Amputation, revascularization, or hospitalization for medical treatment	6.84	9.60	0.215
Mean predicted probability of lower limb amputation	0.06 ± 0.14	0.08 ± 0.16	0.095

*Any one of the cells <3 SD: standard deviation.

**Table 3:** Baseline characteristics and clinical outcomes of diabetic patients with lower extremity arterial stenosis receiving peripheral artery stenting by patients’ monthly income level.

	Monthyly income ≤17,280 (N = 449)	Monthly income >17,280 (N = 210)	P-value
Age (mean ± SD)	72.69 ± 8.04	68.01 ± 9.16	<0.001
Male (%)	68.37	62.38	0.129
*Comorbidities*			
Diabetes-related late complications (%)			
Cardiovascular disease	98.44	95.24	0.016
Ischemic heart disease	65.48	61.43	0.312
Cerebrovascular disease	58.80	49.05	0.019
Ketoacidosis or hyperosmolarity	2.00	1.90	1.000
Retinopathy	32.29	22.86	0.013
Neuropathy	28.73	28.10	0.866
Nephropathy	47.66	55.24	0.070
Myocardial infarction	6.68	9.05	0.281
Ischemic stroke	48.33	43.33	0.231
Chronic renal failure	19.60	27.62	0.021
Chronic liver disease	9.58	12.38	0.273
Chronic lung disease	22.94	21.43	0.665
Depression	7.13	2.86	0.028
Charlson’s index (mean ± SD)	4.05 ± 2.22	4.19 ± 2.36	0.470
Number of different ICD-9 diagnoses (mean ± SD)	21.72 ± 9.88	20.53 ± 9.61	0.146
Number of cardiovascular-related diagnoses (mean ± SD)	4.78 ± 3.00	4.76 ± 3.38	0.936
*Medication use* (*%*)			
Biguanides	64.59	59.52	0.210
Sulfonylurea	71.27	75.24	0.288
Alpha-glucosidase inhibitors	19.15	20.95	0.589
Thiazolidinediones	22.27	18.10	0.220
Glinides	16.93	20.48	0.270
Fast-acting insulins	32.74	31.43	0.737
Basal insulins	11.36	11.90	0.838
Aspirin	79.06	76.19	0.405
Clopidogrel	43.65	47.14	0.401
Cilostazol	24.28	20.48	0.281
Warfarin	6.24	9.52	0.130
ACE inibitors	41.87	42.38	0.902
Angiotensin receptor blockers	54.79	46.67	0.052
Alpha-blockers	25.17	17.14	0.022
Beta-blockers	63.03	60.95	0.608
Calcium channel blockers	77.28	74.76	0.477
Directics	56.12	55.71	0.921
Statins	52.34	52.86	0.901
Fibrates	14.25	17.62	0.264
*Resource utilization* (*mean* ± *SD*)			
Number of A_1_C measurement	2.17 ± 1.91	1.84 ± 1.80	0.035
Number of lipid-related lab test	5.87 ± 5.07	5.15 ± 4.95	0.085
Number of peripheral artery ultrasound examination	0.03 ± 0.19	0.03 ± 0.24	0.797
Number of outpatient visits due to diabetes	19.24 ± 12.89	15.61 ± 9.75	<0.001
Number of outpatient visits not due to diabetes	52.66 ± 29.75	46.36 ± 26.84	0.009
Number of emergency department visit	1.66 ± 2.91	1.10 ± 1.47	0.001
Number of cardiology outpatient visits	5.94 ± 9.18	5.19 ± 8.15	0.311
Number of cardiovascular surgeon outpatient visits	2.47 ± 5.09	2.31 ± 5.18	0.708
Number of cardiovascular-related physician visits	20.84 ± 13.01	16.68 ± 10.32	<0.001
Coronary revascularization %	10.47	11.90	0.582
Prior amputation %	2.45	7.62	0.002
Amputation at index hospitalization %	2.45	2.38	0.957
Number of hospitalization due to diabetes	0.97 ± 1.36	1.05 ± 1.41	0.519
Number of hospitalization not due to diabetes	1.23 ± 1.58	1.37 ± 1.63	0.296
Number of hospitalization due to lower extremity complications	0.14 ± 0.41	0.13 ± 0.50	0.853
Number of hospital days	11.28 ± 23.93	12.35 ± 24.88	0.598
Number of cardiovascular-related hospital days	1.04 ± 1.39	1.11 ± 1.40	0.550
Outcome occurrence (%)			
Amputation	7.13	7.62	0.821
Thigh	*	*	0.440
Foot, ankle, leg	5.35	7.14	0.362
Amputation, revascularization, or hospitalization for medical treatment	8.46	8.57	0.963
Mean predicted probability of lower limb amputation	0.07 ± 0.15	0.08 ± 0.15	0.696

*Any one of the cell <3.

*Standardized mean difference using montly income >17,280 as the reference.

SD: standard deviation

**Table 4:** Baseline characteristics and clinical outcomes of diabetic patients with lower extremity arterial stenosis receiving peripheral artery stenting by regional difference as measured by hospital location in northern and non-northen Taiwan.

	Northern (N = 407)	Non-northern (N = 252)	P-value
Age (mean ± SD)	72.06 ± 8.46	69.81 ± 8.87	0.001
Male (%)	66.34	66.67	0.931
*Comorbidities*			
Diabetes-related late complications (%)			
Cardiovascular disease	98.03	96.43	0.206
Ischemic heart disease	66.09	61.11	0.195
Cerebrovascular disease	55.53	55.95	0.915
Ketoacidosis or hyperosmolarity	2.46	1.19	0.389
Retinopathy	29.98	28.17	0.622
Neuropathy	25.06	34.13	0.012
Nephropathy	47.17	54.76	0.058
Myocardial infarction	7.62	7.14	0.822
Ischemic stroke	46.19	47.62	0.721
Chronic renal failure	20.39	25.00	0.166
Chronic liver disease	7.62	15.08	0.002
Chronic lung disease	22.60	22.22	0.909
Depression	5.90	5.56	0.855
Charlson’s index (mean ± SD)	3.91 ± 2.10	4.38 ± 2.48	0.012
Number of different ICD-9 diagnoses (mean ± SD)	20.46 ± 9.21	22.76 ± 10.55	0.005
Number of cardiovascular-related diagnoses (mean ± SD)	4.73 ± 3.02	4.86 ± 3.28	0.604
*Medication use* (*%*)			
Biguanides	62.41	63.89	0.702
Sulfonylurea	70.52	75.79	0.140
Alpha-glucosidase inhibitors	18.67	21.43	0.388
Thiazolidinediones	21.62	19.84	0.585
Glinides	17.94	18.25	0.918
Fast-acting insulins	28.99	37.70	0.020
Basal insulins	10.81	12.70	0.461
Aspirin	79.61	75.79	0.250
Clopidogrel	42.26	48.81	0.100
Cilostazol	26.54	17.46	0.007
Warfarin	8.11	5.95	0.301
ACE inibitors	39.56	46.03	0.102
Angiotensin receptor blockers	56.02	46.03	0.013
Alpha-blockers	23.10	21.83	0.705
Beta-blockers	61.18	64.29	0.424
Calcium channel blockers	74.69	79.37	0.169
Directics	53.56	59.92	0.110
Statins	53.32	51.19	0.595
Fibrates	16.46	13.49	0.304
*Resource utilization* (*mean* ± *SD*)			
Number of A_1_C measurement	2.25 ± 1.96	1.78 ± 1.72	0.001
Number of lipid-related lab test	5.99 ± 4.97	5.08 ± 5.11	0.025
Number of peripheral artery ultrasound examination	0.04 ± 0.20	0.02 ± 0.22	0.433
Number of outpatient visits due to diabetes	18.78 ± 12.41	16.96 ± 11.50	0.060
Number of outpatient visits not due to diabetes	49.38 ± 28.03	52.70 ± 30.40	0.153
Number of emergency department visit	1.51 ± 2.68	1.44 ± 2.34	0.732
Number of cardiology outpatient visits	5.39 ± 8.06	6.20 ± 10.03	0.275
Number of cardiovascular surgeon outpatient visits	2.39 ± 5.12	2.48 ± 5.12	0.811
Number of cardiovascular-related physician visits	20.53 ± 12.39	17.86 ± 12.17	0.007
Coronary revascularization %	9.83	12.70	0.251
Prior amputation %	3.69	4.76	0.498
Amputation at index hospitalization %	1.97	3.17	0.327
Number of hospitalization due to diabetes	0.95 ± 1.37	1.07 ± 1.39	0.302
Number of hospitalization not due to diabetes	1.21 ± 1.55	1.37 ± 1.67	0.217
Number of hospitalization due to lower extremity complications	0.13 ± 0.45	0.14 ± 0.42	0.805
Number of hospital days	11.77 ± 20.89	11.39 ± 28.84	0.856
Number of cardiovascular-related hospital days	1.06 ± 1.39	1.08 ± 1.41	0.811
Outcome occurrence (%)			
Amputation	5.41	10.32	0.018
Thigh	0.98	1.19	0.999
Foot, ankle, leg	3.93	9.13	0.006
Amputation, revascularization, or hospitalization for medical treatment	6.39	11.90	0.014
Mean predicted probability of lower limb amputation	0.05 ± 0.12	0.10 ± 0.18	<0.001

*Standardized mean difference using non-northern as the reference.

SD: standard deviation.

**Table 5:** Risk differences of amputation, revascularization, or hospitalization for medical treatment comparing diabetic patients receiving peripheral artery stenting (N = 659) vs. bypass surgery (N = 5,652) by instrumental variable analyses^*^.

	Crude analysis		Analysis using calendar year as IV		Analysis using patients’ monthly income level as IV		Analysis using regional difference as IV	
	Risk difference (95% CI)	P value	Risk difference (95% CI)	P value	Risk difference (95% CI)	P value	Risk difference (95% CI)	P value
Amputation	−0.083 (−0.105, −0.061)	<0.0001	−0.003 (−0.053, 0.046)	0.89	0.058 (−0.055, 0.171)	0.31	0.148 (0.068, 0.227)	<0.001
Foot, ankle, leg	−0.015 (−0.023, −0.006)	0.02	−0.009 (−0.030, 0.013)	0.42	−0.009 (−0.058, 0.040)	0.72	0.008 (−0.026, 0.043)	0.64
Thigh	−0.058 (−0.078, −0.038)	<0.0001	0.011 (−0.033, 0.055)	0.63	0.077 (−0.024, 0.177)	0.14	0.121 (0.050, 0.192)	0.001
Amputation, revascularization, or hospitalization for medical treatment	−0.104 (−0.127, −0.080)	<0.0001	−0.027 (−0.080, 0.026)	0.31	0.016 (−0.105, 0.137)	0.79	0.142 (0.057, 0.228)	0.001

*Risk differences at 2-year using bypass surgery as the reference.

IV: instrumental variable.
