# Supplementary material for: Stent revascularization versus bypass surgery for peripheral artery disease in type 2 diabetic patients – an instrumental variable analysis
Source: Sci Rep. 2016 Nov 18;6:37177. doi: 10.1038/srep37177 (PMC5114545; doi:10.1038/srep37177)

Stent revascularization versus bypass surgery for peripheral artery disease in type 2 diabetic patients – an instrumental variable analysis

Authors: Chia-Hsuin Chang; Jou-Wei Lin; Jiun Hsu; Li-Chiu Wu; Mei-Shu Lai

Number of Supplementary Tables: 5

Number of Supplementary Figures: 1

Supplementary Table 1. ICD-9-CM diagnostic codes used to identify patients with diabetes mellitus and co-morbid diseases

Comorbidities	ICD-9-CM codes
Diabetes mellitus	250
Type 1 diabetes mellitus	250.x1, 250.x3
Cardiovascular disease	401-405, 410-414, 425-428, 429.1-429.3, 441, 442
Ischemic heart disease	410-414
Myocardial infarction	410, 412
Cerebrovascular disease	430-438
Ischemic stroke	433, 434, 436 (exclude 800, 801, 802, 803, 804, 850, 851, 852, 853, 854 ,V57)
Ketoacidosis or hyperosmolarity	250.1, 250,2
Retinopathy	250.5, 362.0x, 362.53, 362.83, 364.42, 379.23, 369
Neuropathy	250.6, 337.1, 354, 355, 356.8, 357.2, 358.1, 713.5, 729.2
Nephropathy	250.4, 580-588, 590, 593, 595, 596, 599, 791.0
Chronic kidney disease	403.01, 403.11, 403.91, 404.02, 404.03, 404.12, 404.13, 404.92, 404.93, 585, V45.1, V56.0, V56.8
Chronic liver disease	070.2x, 070.3x, V02.61, 070.41, 070.44, 070.51, 070.54, V02.62, 571.0, 571.1, 571.2, 571.3, 571.4, 571.5, 571.6
Chronic lung disease	490-496, 500-508
Depression	296.2, 296.3, 298.0, 300.4, 309.0, 309.1, 293.83, 296.82, 296.90, 309.28, 311, V79.0

Supplementary Table 2. Percutaneous stents approved for treating ilio-femoral artery stenosis in Taiwan between 1 January 2000 and 31 December 2007

Boston Scientific Wallstent Iliac Endoprosthesis with Unistep Plus Delivery System
Angiomed Luminexx Vascular Stent
Cook Zilver Vascular Stent (Iliac artery)
Angiomed Bard E. Luminexx Vascular Stent

Supplementary Table 3. Medication and the Anatomical Therapeutic Chemical (ATC) classification system

Medication	ATC code
Biguanides	A10BA
Sulfonylurea	A10BB
Alpha-glucosidase inhibitors	A10BF
Thiazolidinediones	A10BG
Glinides	A10BX02, A10BX03
Fast-acting insulins	A10AB
Basal insulins	A10AE01, A10AE04, A10AE05
Aspirin	B01AC06, N02BA01
Clopidogrel	B01AC04
Cilostazol	B01AC23
Wafarin	B01AA03
Angiotensin converting enzyme inhibitors	C09AA
Angiotensin receptor blockers	C09CA
Alpha-blockers	C02CA
Beta-blockers	C07A
Calcium channel blockers	C08C, C08D, C08E
Diuretics	C03
Other anti-hypertensive agents	C02A, C02B, C02CC, C02D
Fibrates	C10AB
Statins	C10AA

Supplementary Table 4. Baseline characteristics of diabetic patients with ilio-femoral artery stenosis during the 12-month period in the propensity score-matched cohort preceding peripheral artery bypass surgery or stenting

	Propensity score-matched cohort		
	Stenting (N = 433)	Bypass surgery (N = 1,196)	Standardized mean difference
Age (mean ± SD)	70.41±9.16	69.49±10.13	-0.10
Male (%)	60.51	53.34	-0.15
Comorbidities			
Cardiovascular disease	34.87	36.29	0.03
Ischemic heart disease	19.40	17.06	-0.06
Cerebrovascular disease	9.01	8.36	-0.02
Ketoacidosis or hyperosmolarity	0.46	0.50	0.01
Retinopathy	2.77	2.76	0.00
Neuropathy	2.08	1.51	-0.04
Nephropathy	17.78	24.50	0.17
Myocardial infarction	3.23	3.51	0.02
Ischemic stroke	31.41	25.33	-0.14
Chronic renal failure	14.09	20.57	0.17
Chronic liver disease	1.85	1.51	-0.03
Chronic lung disease	1.62	1.92	0.02
Depression	0.23	0.17	-0.01
Charlson's index (mean ± SD)	0.96±1.50	1.13±1.62	0.11
Number of different ICD-9 diagnoses (mean ± SD)	2.47±2.88	2.83±3.15	0.12
Number of cardiovascular-related diagnoses (mean ± SD)	0.74±1.29	0.74±1.22	0.00
Medication use (%)			
Biguanides	55.20	50.08	-0.10
Sulfonylurea	67.90	63.38	-0.10
Alpha-glucosidase inhibitors	19.86	19.23	-0.02
Thiazolidinedione	21.71	19.23	-0.06
Glinides	19.86	22.32	0.06
Any oral anti-diabetic agents	80.37	77.09	-0.08
Fast-acting insulin	36.49	42.22	0.12
Basal insulin	12.93	14.13	0.04
Aspirin	72.75	67.14	-0.12
Clopidogrel	32.56	28.01	-0.10
Cilostazol	26.79	28.34	0.03

Warfarin	6.24	5.77	-0.02
ACE inhibitors	43.19	41.89	-0.03
Angiotensin receptor blockers	47.58	46.07	-0.03
Alpha-blockers	18.71	19.06	0.01
Beta-blockers	58.43	57.02	-0.03
Calcium channel blockers	75.06	76.67	0.04
Diuretics	55.66	57.53	0.04
Other anti-hypertensive agents	9.01	10.95	0.06
Statins	46.19	42.89	-0.07
Fibrates	13.63	15.72	0.06
Number of different prescription drugs (mean ± SD)	45.96±24.38	48.37±25.10	0.10
Number of cardiovascular-related medications (mean ± SD)	7.81±5.04	7.86±5.26	0.01
Resource utilization (mean ± SD)			
Number of A_1C measurement	1.93±1.91	1.91±1.81	-0.01
Number of lipid-related lab test	5.24±5.26	5.43±5.22	0.04
Number of peripheral artery ultrasound examination	0.04±0.22	0.05±0.27	0.04
Number of outpatient visits due to diabetes	17.37±11.61	17.15±11.52	-0.02
Number of outpatient visits not due to diabetes	47.99±26.85	48.69±26.77	0.03
Number of emergency department visit	1.45±2.56	1.68±2.40	0.09
Number of cardiology outpatient visits	5.71±9.12	6.11±8.94	0.04
Number of cardiovascular surgery outpatient visits	3.04±5.88	3.24±6.66	0.03
Number of cardiovascular-related physician visits	17.21±11.07	16.10±12.72	-0.09
Coronary revascularization %	8.78	7.78	-0.04
Prior amputation %	5.54	7.94	0.10
Amputation at index hospitalization %	3.70	7.94	0.18
Number of hospitalization due to diabetes	1.06±1.49	1.26±1.58	0.13
Number of hospitalization due to lower extremity complications	0.18±0.49	0.23±0.54	0.10
Number of hospitalization not due to diabetes	1.34±1.72	1.58±1.85	0.13
Number of hospital days	13.27±28.21	16.10±26.34	0.10
Number of cardiovascular-related hospital days	1.06±1.50	1.19±1.57	0.08

SD: standard deviation

Supplementary Table 5. Crude incidence rates and hazard ratios of amputation, revascularization, or hospitalization for medical treatment in propensity score-matched cohort of diabetic patients receiving peripheral artery bypass surgery and stenting

	Incidence rate per 1,000 person-years (95% CI)		Crude Hazard ratio † (95% CI)	P-value	Hazard ratio † (95% CI) adjusted for age, sex, and covariates that were imbalanced ‡	P-value
	Stenting *	Bypass surgery *				
Amputation	57.75 (57.23-58.26)	94.46 (94.03-94.89)	0.65 (0.47-0.89)	0.007	0.67 (0.48-0.92)	0.013
Foot, ankle, leg	47.14 (46.68-47.60)	72.11 (71.74-72.49)	0.70 (0.49-0.99)	0.043	0.72 (0.50-1.03)	0.063
Thigh	8.25 (8.06-8.44)	19.30 (19.10-19.49)	0.45 (0.20-1.01)	0.052	0.47 (0.21-1.06)	0.070
Amputation, revascularization, or hospitalization for medical treatment	63.64 (63.10-64.17)	110.20 (109.74-110.67)	0.61 (0.46-0.83)	0.001	0.63 (0.46-0.85)	0.003

* Stenting: total follow-up 848.55 person-years; Bypass surgery: total follow-up 1969.11 person-years

† Hazard ratio using bypass surgery as the reference

† Including sex, nephropathy, ischemic stroke, chronic renal failure, Charlson's index, number of different ICD-9 diagnoses, fast-acting insulin, aspirin, amputation at index hospitalization, number of hospitalization due to diabetes, and number of hospitalization not due to diabetes

Supplementary Figure

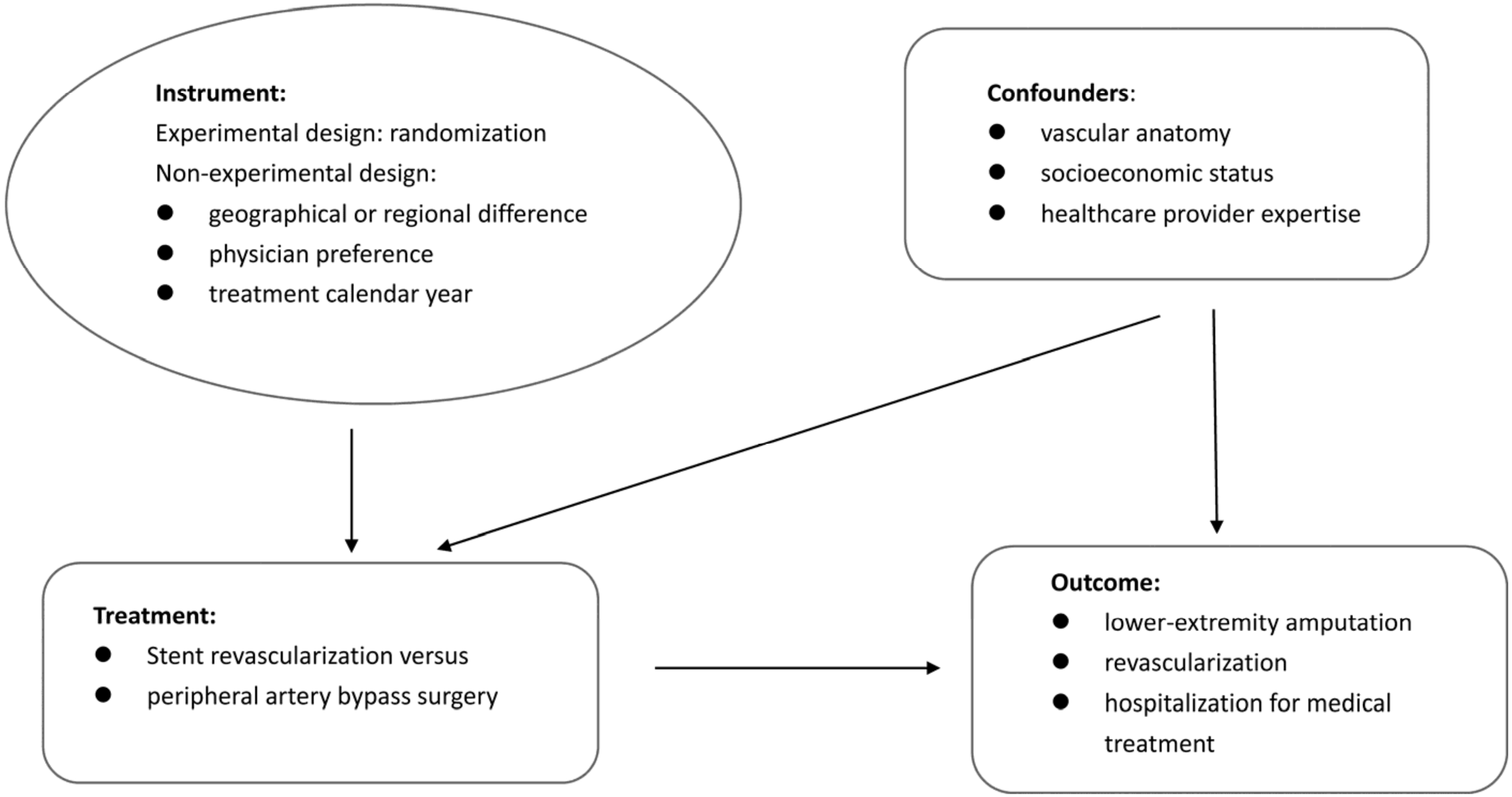

Supplement: Supplementary Information [file srep37177-s1.pdf]
